# Coffee and pancreatic cancer risk among never‐smokers in the UK prospective Million Women Study

**DOI:** 10.1002/ijc.31994

**Published:** 2018-12-28

**Authors:** Charlie D Zhou, Ai Seon Kuan, Gillian K Reeves, Jane Green, Sarah Floud, Valerie Beral, TienYu Owen Yang, on behalf of the Million Women Study Collaborators

**Affiliations:** ^1^ Royal Free London NHS Foundation Trust London NW3 2QG United Kingdom; ^2^ Cancer Epidemiology Unit Nuffield Department of Population Health Oxford OX3 7LF United Kingdom

**Keywords:** coffee, pancreatic neoplasms, smoking, cohort study

## Abstract

Reported associations between coffee consumption and an increased risk of pancreatic cancer could be due to residual confounding by smoking and/or biased recall of coffee consumption in retrospective studies. Studying associations prospectively in never smokers should minimize these problems, but thus far such studies have included relatively small numbers of cases. In our study, 309,797 never‐smoking women self‐reported typical daily coffee consumption at a mean age of 59.5 years (SD 5.0 years) and were followed up for a median of 13.7 years (IQR: 12.2–14.9) through record linkage to national health cancer and death registries. During this period, 962 incident cases of pancreatic cancers were registered. Cox regression was used to calculate adjusted relative risks [RRs] of incident pancreatic cancer with 95% confidence intervals [CIs] in relation to coffee consumption at baseline. After adjustment for potential confounding factors, including body mass index and alcohol consumption, RRs of pancreatic cancer in never‐smokers who reported usually consuming 1–2, 3–4, and ≥ 5 cups of coffee daily, compared to nondrinkers of coffee, were 1.02 (CI 0.83–1.26), 0.96 (0.76–1.22), and 0.87 (0.64–1.18), respectively (trend *p* = 0.2). A meta‐analysis of results from this cohort and 3 smaller prospective studies found little or no statistically significant association between coffee consumption and pancreatic cancer risk in never smokers (summary RR = 1.00, CI 0.86–1.17 for ≥2 *vs*. zero cups of coffee per day).

## Abstract 2

**What's new?**

Reported associations between coffee consumption and pancreatic cancer risk could be due to residual confounding by smoking or recall bias of coffee consumption in retrospective studies. In this large prospective study and meta‐analysis restricted to never‐smokers, we found no significant association between coffee consumption and pancreatic cancer risk.

**Abbreviations**
BMI: body mass index
CI: 95% confidence intervals
ICD‐10: 10th revision of the International Classification of Diseases
IQR: interquartile range
NIH‐AARP: National Institutes of Health‐AARP (formerly the American Association of Retired Persons)
RR: relative risk
SD: standard deviation
UK: United Kingdom
US: United States
USA: United States of America

## Introduction

Initial evidence suggesting an association between coffee consumption and pancreatic cancer risk was from retrospective studies in which information on coffee consumption was collected after cancer diagnosis,^1^, ^2^, ^3^ where selective recall of coffee consumption after cancer diagnosis cannot be excluded. Findings from both retrospective and prospective studies may also be confounded by smoking, as smoking is a strong risk factor for pancreatic cancer^4^, ^5^ and smokers have generally been found to drink more coffee than never‐smokers.^6^, ^7^, ^8^, ^9^, ^10^, ^11^ In many studies, lack of detailed information means that basic adjustment for smoking may be inadequate^12^ and so residual confounding by smoking cannot be distinguished from a modest association with coffee consumption.^13^ None of the prospective studies included in a recent meta‐analysis included more than 300 incident cases of pancreatic cancer,^14^ and there was considerable variation in the extent to which smoking was adjusted for across studies.

Studying the association between coffee consumption and pancreatic cancer prospectively in never‐smokers avoids recall bias and residual confounding from smoking. We report here on the association between self‐reported coffee consumption and risk of pancreatic cancer among a large cohort of United Kingdom (UK) women who had never smoked. We report also the results of a meta‐analysis combining our findings with those from three smaller prospective studies which have published results on coffee consumption and pancreatic cancer risk in never‐smokers.

## Materials and Methods

About 1.3 million UK women born in 1935–1950 were recruited to the Million Women Study in 1996–2001. Participants returned a questionnaire at recruitment answering questions on their health and lifestyle and signed consent for follow‐up through linkage to medical records. Participants were posted another questionnaire 3 years after recruitment to update information including that on smoking, and to provide new information on diet, including coffee consumption. All women were followed using their unique National Health Service (NHS) number and other identifiers to link to the National Health Service Central Registers on cancers and death, and with causes coded to the 10th revision of the International Classification of Diseases (ICD‐10). The study has been described elsewhere^15^ and details of the study questionnaires and of data access are available at the Million Women Study website (www.millionwomenstudy.org). The study is approved by the Oxford and Anglia Multi‐Centre Research and Ethics Committee.

At the three‐year resurvey, women were asked for the first time how many cups of coffee they drank on a typical day. This information was used in these analyses presented and served as baseline for the analysis. The repeatability of the baseline self‐reported coffee intake has been assessed in a random sample of women who provided additional information on coffee consumption 2 years after baseline, when intake was found to be stable.^16^ Furthermore, approximately two thirds of women reported coffee intake in another resurvey 4 years after baseline in which the correlation coefficient with baseline reported coffee consumption was >0.6.^11^

Women were eligible for these analyses if they reported at baseline never having smoked. Women were excluded if they had a prior reported or registered cancer (except non‐melanoma skin cancer); if they had reported a change in diet within the last 5 years because of illness; or if they reported being diagnosed with diabetes in the last 5 years), as this may be a sign of possible subclinical pancreatic cancer,^17^, ^18^ although sensitivity analyses were performed with the inclusion of these women.

Participants were followed from baseline to the date of registration of any malignant cancer (except non‐melanoma skin cancer), death, emigration, loss to follow‐up, or end of available follow‐up (31 December 2015), whichever was the earliest. Incident cases of pancreatic cancer were identified through linkage to the National Health Service Registers by ICD‐10 code C25.

Using Cox regression, we estimated the relative risks (RRs) and 95% confidence intervals (CIs) for incident pancreatic cancer in relation to baseline reported coffee consumption (0, 1–2, 3–4, and ≥ 5 cups/day). In all analyses, non‐coffee drinkers were taken as the reference category, and relative risks were stratified by year of birth and year that they reported coffee consumption. Relative risks were further adjusted for dietary energy intake (fifths), type of meat consumed (none, poultry, red meat, one type of processed meat, two or more types of processed meat), height (<155, 155–159, 160–164, 165–169, 170–174, 175+ cm), body mass index (BMI, <22.5, 22.5–24.9, 25.0–29.9, 30.0–34.9, 35+ kg/m^2^), social deprivation (Townsend deprivation index in fifths), alcohol intake (0, 1–4, 5–14, 15–29, 30+ g/day), educational qualifications (tertiary, secondary, technical & below) and region (Scotland and nine regions in England). Missing values of these adjustment variables were assigned to a separate category. Information on height, social deprivation, educational qualifications and region was from the recruitment questionnaire, whilst information on the remaining variables was from the three‐year survey. Trend estimates (per 1 cup increase daily) across baseline coffee consumption categories were based on the means of coffee consumption in each category reported in the resurvey approximately 4 years later.

Sensitivity analyses, to assess potential effect modification, examined pancreatic cancer risk in relation to coffee consumption in subgroups, classified by alcohol consumption and by their BMI. To assess for bias related to change in reported coffee consumption prior to cancer diagnosis, relative risks were compared in the first 5 years of follow‐up compared to 5+ years follow‐up, and were also estimated for those who reported coffee consumption in another resurvey approximately 4 years later and remained in the same coffee consumption category. Relative risks were also estimated after inclusion of those who had reported a diagnosis of diabetes within the last 5 years at baseline. Other sensitivity analyses were restricted to cases with histologically confirmed pancreatic adenocarcinoma, not otherwise specified, defined by the International Classification of Diseases Morphology of Neoplasms, 3rd Edition code M81403.

A systematic review was conducted for published prospective studies which reported the association between coffee consumption and pancreatic cancer risk among never‐smokers. We searched for articles published in or prior to December 2017 from EMBase and PubMed using a combination of keywords related to coffee and pancreatic cancer (coffee AND (pancrea* cancer OR pancrea* neoplas* OR pancrea* carcinoma OR pancrea* malignan*), and combined study‐specific RRs using inverse‐variance weighting. Generalised least squares were used for combining categories within each study. All statistical analyses were performed using Stata/MP 14.1 (StataCorp, College Station, TX, USA).

## Results

In total 381,928 never‐smokers reported their daily coffee consumption at baseline, and for the analyses we excluded 19,670 with prior cancer, 49,769 who had changed their diet due to illness, and 2,692 with a recent diagnosis of diabetes. The remaining 309,797 never‐smokers were followed from the mean age of 59.5 years (standard deviation [SD] 5.0 years) for 13.7 (IQR: 12.2–14.9) years on average (Table 1). The mean coffee consumption reported at baseline was 2.1 (SD 1.8) cups/day, with 12% reporting that they did not drink coffee daily. In 202,134 women (65%) for whom repeat measures of coffee consumption 4 years after baseline was available, the repeated measures in the baseline categories were similar to the baseline measures, with some regression to the mean (Table 1). Among the never‐smokers there were no major differences between coffee drinkers and non‐drinkers in age, height, alcohol consumption, dietary energy intake, frequency of processed meat intake, social deprivation, education and average duration of follow up (Table 1).

**Table 1:** Characteristics of never smokers in the Million Women Study, by daily coffee consumption provided at 3‐year study resurvey (analysis baseline)

			Daily coffee consumption (cups/day)		
Characteristics at analysis baseline	All women	Non‐coffee drinkers	1–2	3–4	≥5
Number of women	309,797	36,822	172,481	74,122	26,372
Daily coffee intake					
Reported at baseline cups (mean, SD)	2.1 (1.8)	0.0 (0.0)	1.5 (0.5)	3.4 (0.5)	6.1 (2.2)
Reported approximately 4 years later (mean, SD)	2.3 (2.5)	0.4 (1.0)	1.7 (1.7)	3.3 (2.7)	5.1 (3.9)
Age at the start of follow‐up, years (mean, SD)	59.5 (5.0)	58.8 (4.9)	60.0 (5.1)	59.2 (4.8)	58.6 (4.6)
Lowest fifth of socioeconomic status, *n* (%)^1^	61,431 (20.0)	8,490 (23.2)	33,881 (19.8)	13,497 (18.3)	5,563 (21.2)
With any educational qualification, *n* (%)^1^	58,419 (19.1)	7,281 (20.0)	30,972 (18.2)	15,193 (20.8)	4,973 (19.2)
Height, cm (mean, SD)^1^	162.5 (6.5)	162.2 (6.6)	162.5 (6.5)	162.6 (6.5)	162.5 (6.5)
Body mass index, kg/m^2^ (mean, SD)	25.7 (4.3)	25.6 (4.4)	25.5 (4.1)	26.0 (4.3)	26.6 (4.7)
Daily alcohol intake, grams (mean, SD)	4.8 (6.5)	3.8 (6.3)	4.6 (6.4)	5.4 (6.8)	5.0 (6.8)
Daily dietary energy intake, kcal (mean, SD)	1,660 (427)	1,633 (432)	1,662 (423)	1,661 (422)	1,684 (453)
Processed meat intake at least once weekly, *n* (%)	201,051 (65.3)	22,287 (61.0)	113,933 (66.4)	48,098 (65.3)	16,733 (63.8)
Follow‐up in years (median, IQR)	13.7 (12.2–14.9)	13.6 (12.2–14.9)	13.7 (12.2–14.9)	13.7 (12.3–14.9)	13.7 (12.5–14.9)

1 Characteristics at study recruitment (not available at analysis baseline).

During follow‐up, 962 incident cases of pancreatic cancer were registered in never‐smokers. In minimally adjusted analyses, adjusted for year of birth and calendar year at baseline only, there was no significant difference in relative risk of pancreatic cancer in women never‐smokers across all 4 categories of coffee consumption (heterogeneity *p* = 0.7, Table 2). These relative risks were little changed after further multivariate adjustment for region of residence, dietary energy intake, types of meat consumed, height, socioeconomic status, educational qualifications, BMI and alcohol consumption (heterogeneity *p* = 0.6, Table 2). Compared to women who did not drink coffee, adjusted RRs were 1.02 (CI 0.83–1.26) for women who drank 1–2 cups of coffee daily, 0.96 (CI 0.76–1.22) for women who drank 3–4 cups of coffee daily, and 0.87 (CI 0.64–1.18) for women who drank 5 or more cups of coffee daily. There was no significant trend for pancreatic cancer risk with increasing coffee consumption (RR = 0.97, CI 0.92–1.02 increased per cup; trend *p* = 0.2).

**Table 2:** Relative risk (RR) and 95% confidence interval (95%CI) of pancreatic cancer by daily coffee consumption in never smokers in the Million Women Study

Coffee consumption (cups/day)	Number of women	Number of cancer cases	Crude RR^1^	Adjusted RR^2^ (95% CI)
**0**	36,822	106	1.00 (reference group)	1.00 (reference group)
**1–2**	172,481	571	1.00	1.02 (0.83–1.26)
**3–4**	74,122	217	0.95	0.96 (0.76–1.22)
**≥5**	26,372	68	0.88	0.87 (0.64–1.18)
			P for trend = 0.2	P for trend = 0.2
			P for heterogeneity = 0.7	P for heterogeneity = 0.6

1 Relative risks were stratified by year of birth and year at analysis baseline only, with follow‐up time as the underlying time variable.

2 Relative risks were further adjusted for dietary energy intake, type of meat consumed, height, body mass index, social deprivation, alcohol intake, education, and region.

We examined the association with coffee consumption separately in three groups of women subdivided by their BMI (under 25.0 kg/m^2^, 25.0–29.9 kg/m^2^, and 30.0 kg/m^2^ and greater) and three groups subdivided by their alcohol consumption (non‐drinker, 1–14 g alcohol per day, and 15 g or more per day), and found little evidence of effect modification (for modification by BMI *p* = 0.6, for modification by alcohol consumption *p* = 0.2; Appendix Table A1). Nor was there a significant difference in the relative risk in the first 5 years of follow‐up compared to 5+ years of follow‐up (*p* for difference = 0.2; Appendix Table A2). Similarly, we did not find a statistically significant association when we restricted analysis to women who reported coffee consumption within the same category in a follow‐up survey on average 4 years after baseline (Appendix Table A3). Inclusion of the 2,692 women who reported having a recent diagnosis of diabetes also did not change the results substantially (Appendix Table A4). In 962 registered pancreatic cancer cases, specific histology was recorded in 577 cases, among which 477 cases were adenocarcinoma, not otherwise specified. Restriction of our analysis to this registered histology did not substantially change the reported association (Appendix Table A5).

Our systematic review identified three other published prospective studies (all from the US) that have reported the association between coffee consumption and risk of pancreatic cancer among never‐smokers (Appendix Table A6). Two of these studies were relatively small, with less than 100 incident pancreatic cancer cases in each.^19^, ^20^ In both of these studies, the highest category of coffee consumption was relatively modest, at ≥2 cups/day and > 17.5 cups/week (i.e. >2.5 cups/day), respectively. The third study (NIH‐AARP) was larger with 399 pancreatic cancer cases among never‐smokers, and the highest consumption category was ≥4 cups/day.^10^ Because of the variation in coffee consumption categories between studies, we conducted a meta‐analysis across the 3 previous studies for pancreatic cancer risk associated with drinking 2 or more cups of coffee *vs*. non‐drinkers, yielding a summary relative risk of 1.04 (CI 0.82–1.31); with inclusion of the findings presented here the summary relative risk was 1.00 (CI 0.86–1.17; Appendix A7, Appendix A8). For higher levels of coffee consumption, we combined results for the 31 cases who drank 4 cups or more daily in the US NIH‐AARP study and 153 cases who drank 4 cups or more daily in the data presented here (RR = 0.92, CI 0.72–1.18), yielding a combined relative risk of 0.93 (CI 0.75–1.15) compared to non‐drinkers (Appendix A7, Appendix A8).

## Discussion

In this large cohort of female UK never‐smokers, with 962 incident cases of pancreatic cancer after follow‐up of 13.7 years, we found little or no evidence for an association between coffee consumption and pancreatic cancer risk. Combining the findings from our study with those from three other prospective studies of never smokers also showed no significant association of pancreatic cancer risk either with moderate coffee consumption (around 2 cups per day or higher) or higher coffee consumption (4 cups per day or higher).

Smoking is a strong risk factor for pancreatic cancer^4^, ^5^ and has been reported to be associated with coffee consumption in many studies.^6^, ^7^, ^8^, ^9^, ^10^, ^11^ In this cohort, current smokers comprised 26% of those who reported drinking 5 or more cups of coffee per day compared to 11% in those who did not drink coffee.^11^ While residual confounding from smoking cannot be excluded in studies that adjusted for smoking,^12^ prospective investigations among never‐smokers are unconfounded by smoking. Of three previous such investigations,^8^, ^19^, ^20^ only one included more than 100 incident pancreatic cancer cases during follow‐up (the NIH‐AARP study, with 399 cases).^10^ No significant association between coffee consumption and pancreatic cancer incidence was found in any of the three studies.

As well as being restricted to never‐smokers, our analysis accounted for major known potential confounding factors and potential effect modifiers^13^, ^21^ including socio‐economic status, BMI, alcohol and type of meat consumption.^22^ Since our results were not substantially changed after adjustment for these factors and were not significantly modified by these factors, any residual confounding by these factors is likely to be small. All our analyses were mainly based on self‐reported coffee consumption at baseline. In this cohort there is good repeatability of self‐reported consumption over 2 and 4 years after baseline,^11^, ^16^ but changes in consumption or cup volume over a longer period of time or due to variation of cup volume cannot be excluded. We could not examine whether there was any association with the content of the coffee or how the coffee was made or consumed. While coffee consumption may be changed by pre‐diagnostic symptoms of pancreatic cancer, we excluded from the analyses women who reported change of diet due to ill health and those who reported having newly discovered diabetes. We also conducted sensitivity analyses comparing the associations in the first 5 years of follow‐up and after 5+ years follow‐up, but associations of the two periods were not statistically different.

All cancer cases were identified through record linkage to national cancer registries which are known to be complete and reliable,^23^, ^24^ but information on how the individual cases were diagnosed is unavailable.^25^, ^26^, ^27^ Results restricted to the 477 cases registered as histologically‐confirmed adenocarcinoma did not materially change the results.

In this large cohort of women never‐smokers, we found no significant evidence for an association between coffee consumption and risk of pancreatic cancer among women who had never smoked. With three other prospective studies of never‐smokers, the existing evidence does not suggest an association with either moderate or higher coffee consumption.

## Authors’ contribution

VB, JG, GKR and SF conceived and designed the Million Women Study. CDZ, ASK, GKR, JG, VB and TOY conceived and designed the analysis. CDZ and ASK conducted statistical analysis under the primary supervision of TOY. All authors helped interpret the findings. CDZ, ASK and TOY wrote the first draft of the study. GKR, JG, VB, and SF contributed toward subsequent revisions and approved the submitted study.

## Appendix A 1

**Table Appendix A1:** Relative risk (RR) and 95% confidence interval (CI) of pancreatic cancer by daily coffee consumption in never smokers in the Million Women Study, stratified by alcohol consumption and body mass index

Alcohol intake/day	Non‐drinkers		1‐14 g		≥15 g	
Coffee consumption (cups/day)	Number of cancer cases	Adjusted RR^1^ (95% CI)	Number of cancer cases	Adjusted RR^1^ (95% CI)	Number of cancer cases	Adjusted RR^1^ (95% CI)
0	63	1.00 (reference group)	37	0.86 (0.57–1.29)	6	0.97 (0.42–2.25)
1–2	281	1.03 (0.79–1.36)	255	0.89 (0.68–1.18)	35	0.87 (0.57–1.32)
3–4	80	0.84 (0.60–1.16)	120	0.96 (0.70–1.30)	17	0.79 (0.46–1.36)
≥5	33	0.87 (0.57–1.33)	30	0.77 (0.50–1.19)	5	0.76 (0.30–1.89)
		*p* for trend = 0.2		*p* for trend = 0.9		*p* for trend = 0.6
		*p* for heterogeneity = 0.3		*p* for heterogeneity = 0.7		*p* for heterogeneity = 1.0

Body mass index (kg/m^2^)	<25		25–29.9		≥30	
Coffee consumption (cups/day)	Number of cancer cases	Adjusted RR^1^ (95% CI)	Number of cancer cases	Adjusted RR^1^ (95% CI)	Number of cancer cases	Adjusted RR^1^ (95% CI)
0	46	1.00 (reference group)	39	1.24 (0.81–1.90)	11	0.89 (0.46–1.73)
1–2	231	0.97 (0.71–1.33)	205	1.16 (0.84–1.60)	87	1.49 (1.04–2.14)
3–4	89	1.01 (0.71–1.45)	79	1.09 (0.75–1.57)	34	1.24 (0.79–1.93)
≥5	19	0.72 (0.42–1.23)	30	1.19 (0.75–1.88)	12	0.97 (0.51–1.83)
		*p* for trend = 0.4		*p* for trend = 0.7		*p* for trend = 0.5
		*p* for heterogeneity = 0.6		*p* for heterogeneity = 0.9		*p* for heterogeneity = 0.2

*p* for modification by BMI = 0.6; p for modification by alcohol consumption = 0.2.

1 Relative risks were stratified by year of birth and year at analysis baseline with follow‐up time as the underlying time variable and adjusted for dietary energy intake, type of meat consumed, height, body mass index, social deprivation, alcohol intake, education, and region, as appropriate.

**Table Appendix A2:** Relative risk (RR) and 95% confidence interval (95%CI) of pancreatic cancer by daily coffee consumption in never smokers in the Million Women Study, stratified by follow‐up time

	First 5 years of follow‐up		≥5 years of follow‐up	
Coffee consumption (cups/day)	Number of cancer cases	Adjusted RR^1^ (95% CI)	Number of cancer cases	Adjusted RR^1^ (95% CI)
0	30	1.00 (reference group)	76	1.00 (reference group)
1–2	127	0.81 (0.54–1.21)	444	1.10 (0.86–1.41)
3–4	61	0.98 (0.63–1.52)	156	0.96 (0.73–1.26)
≥5	16	0.77 (0.42–1.41)	52	0.92 (0.64–1.30)
		*p* for trend = 0.9		*p* for trend = 0.2
		*p* for heterogeneity = 0.5		*p* for heterogeneity = 0.3

1 Relative risks were stratified by year of birth and year at analysis baseline with follow‐up time as the underlying time variable, and adjusted for dietary energy intake, type of meat consumed, height, body mass index, social deprivation, alcohol intake, education, and region. The association between coffee and pancreatic cancer risk is not different by follow‐up time (<5, ≥5 years), *p* for difference = 0.2.

**Table Appendix A3:** Relative risk (RR) and 95% confidence interval (95%CI) of pancreatic cancer by daily coffee consumption (restricted to women remained in the same coffee intake category at analysis baseline and the subsequent resurvey) in never smokers in the Million Women Study

Coffee consumption (cups/day)	Number of women^1^	Number of cancer cases	Adjusted RR^2^ (95% CI)
0	13,611	22	1.00 (reference group)
1–2	88,233	222	1.35 (0.87–2.10)
3–4	26,677	70	1.49 (0.92–2.42)
≥5	9,313	17	1.05 (0.56–1.99)
			*p* for trend = 0.5
			*p* for heterogeneity = 0.3
≥3	47,646	113	1.36 (0.86–2.15)

1 Of 196,527 women provided information on coffee consumption at both the analysis baseline and the subsequent resurvey, 137,834 remained in the same coffee intake category (0, 1–2, 3–4, 5+ cups/day) in the subsequent resurvey. Of the 18,388 women who reported as non‐drinker at baseline, 74% (*n* = 13,611) still reported as non‐drinker at resurvey. The corresponding percentages were 81% (88,233 in 109,631) for still reporting 1–2 cups daily, 53% (26,677 in 50,797) for still reporting 3–4 cups daily, and 53% (9,313 in 17,711) for still reporting 5 or more cups daily. Of the 68,508 women who reported drinking 3 or more cups of coffee (i.e. either 3–4 cups or 5 or more cups) at analysis baseline and provided repeated information, 47,646 (70%) still reported drinking 3 or more cups daily.

2 Relative risks were stratified by year of birth and year at analysis baseline with follow‐up time as the underlying time variable, and adjusted for dietary energy intake, type of meat consumed, height, body mass index, social deprivation, alcohol intake, education, and region.

**Table Appendix A4:** Relative risk (RR) and 95% confidence interval (95%CI) of pancreatic cancer by daily coffee consumption in never smokers, including women with a recent diagnosis of diabetes

Coffee consumption (cups/day)	Number of cancer cases	Adjusted RR^1^ (95% CI)
0	106	1.00 (reference group)
1–2	582	1.04 (0.84–1.28)
3–4	222	0.99 (0.78–1.25)
≥5	70	0.90 (0.66–1.22)
		*p* for trend = 0.3
		*p* for heterogeneity = 0.7

1 Relative risks were stratified by year of birth and year at analysis baseline with follow‐up time as the underlying time variable, and adjusted for dietary energy intake, type of meat consumed, height, body mass index, social deprivation, alcohol intake, education, and region.

**Table Appendix A5:** Relative risk (RR) and 95% confidence interval (95%CI) of pancreatic cancer restricted to adenocarcinoma, not otherwise specified, by daily coffee consumption in never smokers in the Million Women Study

Coffee consumption (cups/day)	Number of cancer cases	Adjusted RR^1^ (95% CI)
0	60	1.00 (reference group)
1–2	267	0.88 (0.66–1.17)
3–4	120	0.96 (0.70–1.31)
≥5	30	0.68 (0.44–1.05)
		*p* for trend = 0.3
		*p* for heterogeneity = 0.3

1 Relative risks were stratified by year of birth and year at analysis baseline with follow‐up time as the underlying time variable, and adjusted for dietary energy intake, type of meat consumed, height, body mass index, social deprivation, alcohol intake, education, and region.

**Table Appendix A6:** Studies included in meta‐analysis reporting the association between coffee consumption and pancreatic cancer risk among never‐smokers

Study (author, year of publication and country)	Cases	Cohort info	Adjustment	All participants adjusted for smoking		Non‐smokers only	
				Relative risks		Relative risks	
Iowa Women's Health Study (Harnack *et al*. 1997, USA)	66 cancers, 38 never‐smokers	41,837 women, aged 55–69 were recruited in the 1985 Iowa state driver's licence list. Cases were identified by linkage with the State Health Registry of Iowa.	Age, alcohol, smoking	0–7 cups/week 8–17.5 cups/week >17.5 cups/week	1 (reference) 1.82 (0.87–3.82) 2.15 (1.01–4.07)	0–7 cups/week 8–17.5 cups/week ≥17.5 cups/week	1 (reference) 1.36 (0.58–3.20) 1.74 (0.80–3.80)
Health Professionals Follow‐up Study and Nurses’ Health Study I (Michaud *et al*. 2001, USA)	288 cancers, less than 100 never‐smokers	47,794 men were followed up until 1998 and 88,799 women were followed up until 1996. Cases were self‐reported at diagnosis or determined by death certification.	Age, smoking, BMI, diabetes, cholecystectomy, energy intake and period	None < 1 cups/day 1 cups/day 2–3 cups/day > 3 cups/day	1 (reference) 0.94 (0.65–1.36) 0.60 (0.38–0.94) 0.88 (0.65–1.21) 0.62 (0.27–1.43)	None < 1 cups/day 1 cups/day 2–3 cups/day	1 (reference) 1.25 0.72 1.01 (0.61–1.66)
NIH‐AARP Diet and Health Study (Guertin *et al*. 2015, USA)	1,541 cancers, 399 never‐smokers	457,366 participants aged 50–71 and resident in one of six US states or two metropolitan areas and followed up until 2006. Cases were identified by linkage to 11 state cancer registries and the National Death Index.	Age, sex, smoking, diabetes, Ethnic group, BMI, level of education, alcohol, health status, nutritional supplements, marital status, physical activity, history of cardiovascular disease, family history of cancer, total energy intake, nutrient density‐adjusted intake of fruits, vegetables, folate, protein, saturated fat, total fat.	None < 1 cups/day 1 cups/day 2–3 cups/day 4–5 cups/day ≥ 6 cups/day	1 (reference) 1.05 (0.84–1.30) 1.06 (0.86–1.31) 1.03 (0.85–1.26) 1.01 (0.80–1.27) 1.26 (0.94–1.69)	None < 1 cups/day 2–3 cups/day ≥ 4 cups/day	1 (reference) 0.95 (0.71–1.26) 0.98 (0.73–1.31) 0.97 (0.63–1.48)

**Table Appendix A7:** Relative risk (RR) and 95% confidence interval (95%CI) of pancreatic cancer by daily coffee consumption (0, 1+ cups/day) (0, <2, 2+ cups per day) in never smokers in the Million Women Study

**1+ cups/day vs. non‐drinker**		
**Coffee consumption (cups/day)**	**Number of cancer cases**	**Adjusted RR** 1 **(95% CI)**
0	106	1.00 (reference group)
≥1	856	0.99 (0.81–1.22)
		*p* for heterogeneity = 0.9
**>=2 cups/day and <2 cups/day vs. non‐drinker**		
**Coffee consumption (cups/day)**	**Number of cancer cases**	**Adjusted RR** 1 **(95% CI)**
0	106	1.00 (reference group)
<2	320	1.03 (0.82–1.28)
≥2	536	0.97 (0.79–1.20)
		*p* for heterogeneity = 0.7

1 Relative risks were stratified by year of birth and year at analysis baseline with follow‐up time as the underlying time variable, and adjusted for dietary energy intake, type of meat consumed, height, body mass index, social deprivation, alcohol intake, education, and region.

**Table Appendix A8:** Meta‐analysis of prospective reports on the association between coffee consumption and pancreatic cancer risk among never‐smokers

Daily coffee consumption	Type of comparisons	Number of exposed cases	RR (95% CI)
*2+ cups vs. 0–1 cups/day*			
*Study, year reported*			
Iowa Women's Health Study, 1997	>2.5 *vs*. 0–1 (cups/day)	17	1.74 (0.80–3.80)
HPFS & NHS I, 2001	≥2 *vs*. 0 (cups/day)	not available	1.01 (0.61–1.66)
NIH‐AARP Study, 2015	≥2 *vs*. 0 (cups/day)	172	0.98 (0.74–1.30)
Million Women Study, 2018	≥2 *vs*. 0 (cups/day)	536	0.97 (0.79–1.20)
Iowa + HPFS/NHS 1 + NIH‐AARP		>189	1.04 (0.82–1.31)
ALL 4 STUDIES		>725	1.00 (0.86–1.17)
*4+ cups/day vs. none*			
*Study, year reported*			
NIH‐AARP Study, 2015	≥4 *vs*. 0 (cups/day)	31	0.97 (0.63–1.48)
Million Women Study, 2018	≥4 *vs*. 0 (cups/day)	153	0.92 (0.72–1.18)
ALL STUDIES		184	0.93 (0.75–1.15)
